# Novel method of differentiating human induced pluripotent stem cells to mature cardiomyocytes via Sfrp2

**DOI:** 10.1038/s41598-023-31144-3

**Published:** 2023-03-09

**Authors:** Ying-Chang Hsueh, Richard E. Pratt, Victor J. Dzau, Conrad P. Hodgkinson

**Affiliations:** Mandel Center for Heart and Vascular Research, and the Duke Cardiovascular Research Center, Duke University Medical Center, CaRL Building, 213 Research Drive, Durham, NC 27710 USA

**Keywords:** Cell biology, Stem cells

## Abstract

Current methods to generate cardiomyocytes from induced pluripotent stem cells (iPSc) utilize broad-spectrum pharmacological inhibitors. These methods give rise to cardiomyocytes which are typically immature. Since we have recently demonstrated that cardiomyogenesis in vitro and in vivo requires Sfrp2, we asked if Sfrp2 would drive differentiation of human iPSc into cardiomyocytes. Indeed, we found that Sfrp2 induced robust cardiac differentiation. Importantly, replacement of broad spectrum pharmacological inhibitors with Sfrp2 gave rise to mature cardiomyocytes as evidenced by their sarcomere structure, electrophysiological profiles, and ability to form gap junctions.

## Introduction

Coronary artery occlusion causes myocardial necrosis, leading to heart failure and death. Cardiomyocytes-derived from induced pluripotent stem cells (iPSc) are a potential cell source to replace heart tissue lost after infarction^1–3^. Moreover, iPSc-derived cardiomyocytes are a potentially important platform for drug screening^4,5^. The most efficient method to generate iPSc-derived cardiomyocytes adopts a two-staged approach. Initially, iPSCs are treated with a Glycogen Synthase Kinase (GSK) inhibitor^6^ to induce the β-Catenin pathway which drives iPSC conversion to cardiac progenitors. The resulting cardiac progenitors are then differentiated into cardiomyocytes by means of broad-spectrum pharmacological agents which non-discriminately target all Wnts. This protocol gives rise to a high number of cardiomyocytes. However, the phenotype is typically fetal-like which creates a significant issue for in vivo cell therapy as the immature cardiomyocytes can cause arrhythmia after implantation^7–9^.

Several methods have been used in attempt to improve the maturation of iPSc-derived cardiomyocytes. These methods include mechanical stimuli, 3D-structures, chemical factors, as well as genetic regulation^10,11^. However, these methods are only applied after differentiation is attained, requiring specialized equipment or materials and prolonged culture to obtain adult-like cardiomyocytes. Consequently, at present, the current differentiation protocols are unable to produce mature cardiomyocytes in a relatively short period^10^.

We hypothesized that the inability of current differentiation protocols to produce mature cardiomyocytes was due to the indiscriminate nature of broad-spectrum pharmacological inhibitors. Recently, we have demonstrated that mature cardiomyogenesis is induced in vivo by Secreted Frizzled Related Protein 2 (Sfrp2)^12,13^. Consequently, we wanted to determine if replacement of broad-spectrum pharmacological Wnt inhibitors by Sfrp2 would give rise to mature iPSc-derived cardiomyocytes. Indeed, our data showed that Sfrp2 induced significant maturation as evidenced by sarcomere structure, electrophysiological profiles and gap-junction formation.

## Results

### Sfrp2 promotes iPSc differentiation to cardiomyocytes

**Figure 1:**
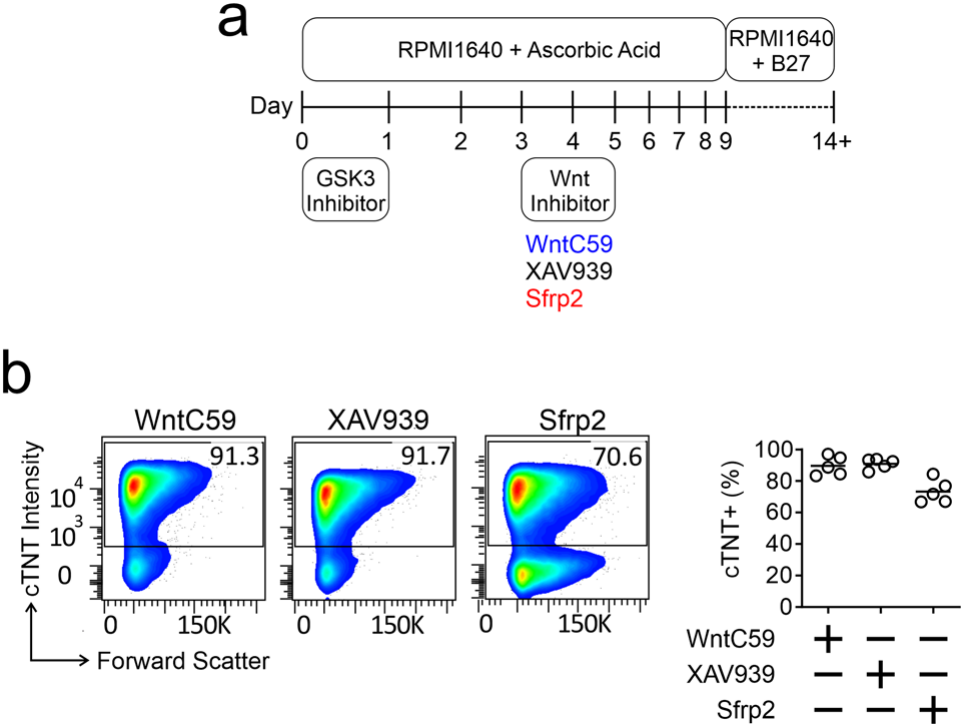
Sfrp2 induced cardiac differentiation. (**a**) Schematic of cardiac differentiation from human iPSc. (**b**) Cardiac differentiation was determined by flow cytometry of the cardiomyocyte marker cardiac troponin-T (cTnT). Cells were analyzed on day 14 of the protocol shown in (a). *N* = 5.

Human iPScs were differentiated into cardiomyocytes via a standard protocol^6^. The protocol requires the initial addition of a GSK3B inhibitor (e.g. CHIR99021, 5 µM) followed by the later addition of a broad-spectrum Wnt pharmacological inhibitor (Fig. 1a). In this study, we used two broad-spectrum Wnt pharmacological inhibitors: WntC59 and XAV939. To address our hypothesis, in addition to the broad-spectrum Wnt pharmacological inhibitors, we also tested a more selective Wnt inhibitor, Sfrp2 (Fig. 1a). The WntC59 and XAV939 doses are those typically employed for iPSc differentiation into cardiomyocytes. The Sfrp2 dose was chosen based on a dose response curve which showed that the EC100 is 1 nM.

In the first instance, cardiomyocyte differentiation was analyzed by flow cytometry of cardiac troponin-T (cTnT) immunostained cells. As shown in Fig. 1b, Sfrp2 induced robust cardiac differentiation from iPSc (~ 70.6%).

### Sfrp2 promotes iPSc-derived cardiomyocyte maturation

**Figure 2:**
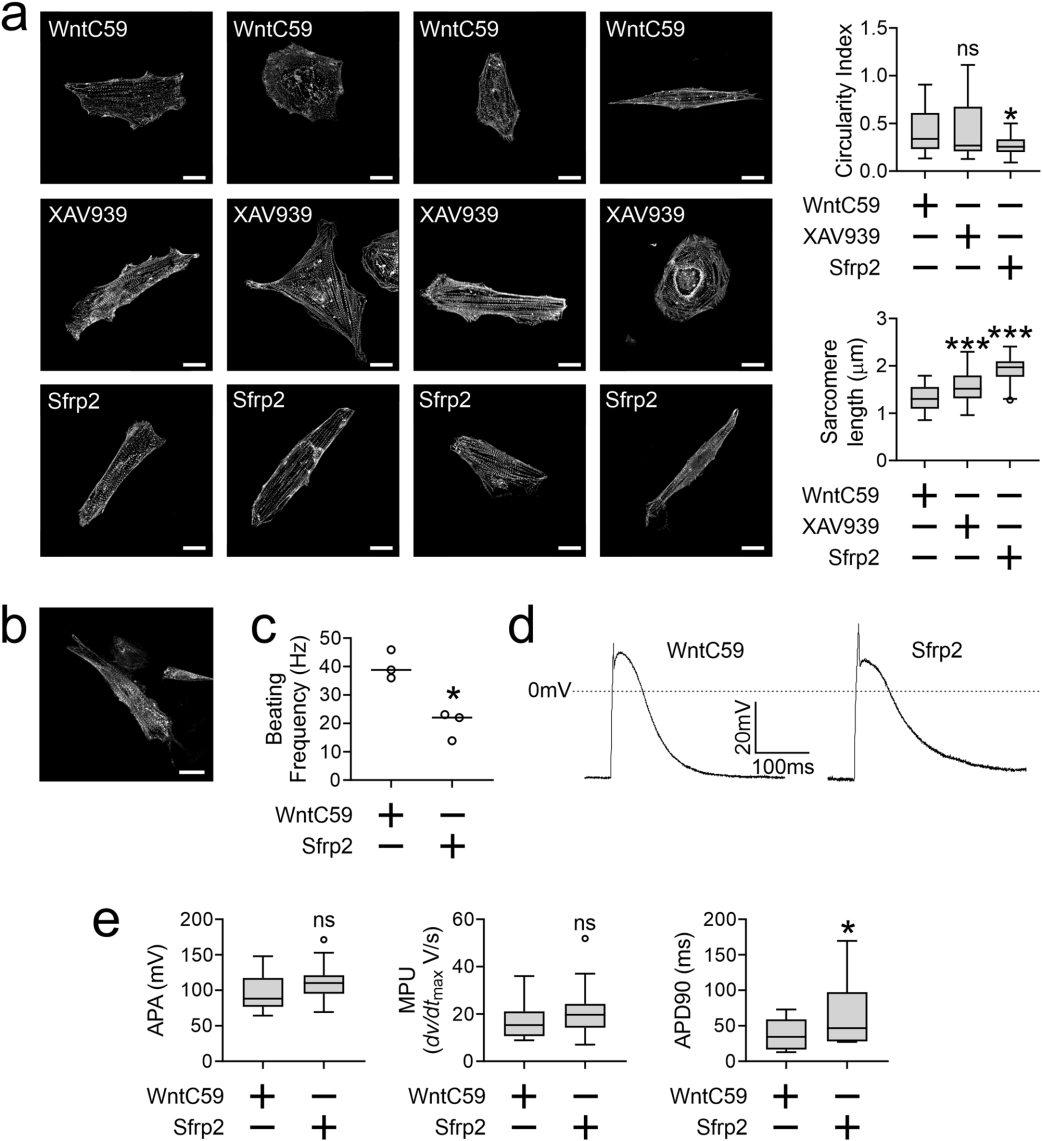
Sfrp2 improves iPSc-derived cardiomyocyte maturation. iPSc were differentiated into cardiomyocytes via the standard broad-spectrum Wnt inhibitor (WntC59, XAV939) method or via the Sfrp2 method. Cells were analyzed on day 14 of differentiation protocol. (**a**) cells were fixed and stained with an antibody targeting α-Actinin (Actn2). *Left hand side:* Representative images shown (scale bar 20 microns). *Right hand side:* Quantification of two parameters of maturation: circularity and sarcomere length. *N* > 30 for each group. Comparisons are made to the WntC59 group: ***P < 0.001, *P < 0.05, ns-not significant. (**b**) Representative image of a Sfrp2-derived cardiomyocyte after 40 days in culture. Scale bar 20 microns. *N* = 3. (**c**) Beating frequency was determined on day-14 of the differentiation protocol by measuring the number of contractions over a 20 s period. For each experiment, 3 wells were analyzed. Representative experiment shown. *P < 0.05. (**d**) Fourteen days after the start of differentiation, patch-clamp was performed. Sample action potentials of cardiomyocytes generated via the standard broad-spectrum Wnt inhibitor (WntC59) approach or via Sfrp2 protocol. (**e**) Patch-clamp data was analyzed for action potential amplitude (APA), maximal rate of depolarization (MPU) (*dv/dt*_*max*_ ( and action potential duration (APD) at 90% of APA. *N* = 15 (WntC59) or 19 (Sfrp2). Comparisons were made to the WntC59 group: *P < 0.05, ns – not significant.

Following the discovery that Sfrp2 induced iPSc to differentiate into cardiomyocytes, we next determined the maturity of these cells compared to cells derived from broad-spectrum Wnt inhibitor treated iPScs. Sarcomere structure is different between immature and mature cardiomyocytes. In immature cardiomyocytes, sarcomeres are patchy. In contrast, in mature cardiomyocytes sarcomeres are present across the entire cell. Moreover, sarcomere length is also longer^14^. To assess sarcomere structure, cardiomyocytes were stained with an α-Actinin (Actn2) antibody. When compared to the standard protocol, as evidenced by more coverage across the cell, sarcomere structure was more mature under the Sfrp2 protocol (Fig. 2a). We chose to analyze the immunostaining for two markers of maturation: circularity and sarcomere length. As cardiomyocytes mature they become less circular^15^. In addition, sarcomeres lengthen^15^. When compared to standard protocols using broad spectrum Wnt inhibitors, Sfrp2 was found to reduce cardiomyocyte circularity and lengthen sarcomeres (Fig. 2a). This result suggested that Sfrp2 gave rise to more mature cardiomyocytes. The phenotype appeared to be stable as up to 40 days in culture there was no evidence of de-differentiation (Fig. 2b). Maturation of ventricular cardiomyocytes is associated with reduced beating frequency^15^. Indeed, when compared to broad-spectrum Wnt inhibitor WntC59, Sfrp2-derived cardiomyocytes were found to have a lower beating frequency (Fig. 2c). To further assess maturation, whole-cell patch clamp was employed. Sample traces indicated that the iPSc-derived cardiomyocytes were physiologically normal (Fig. 2d)^14,16–20^. Analysis of the traces indicated that while action potential amplitude (APA) and *dv/dt*_max_ were similar in both groups, the decay of the action potential (APD90) was significantly longer in Sfrp2-derived cardiomyocytes (Fig. 2e). Lengthened action potential duration is a feature of cardiomyocyte maturation^15^.

**Figure 3:**
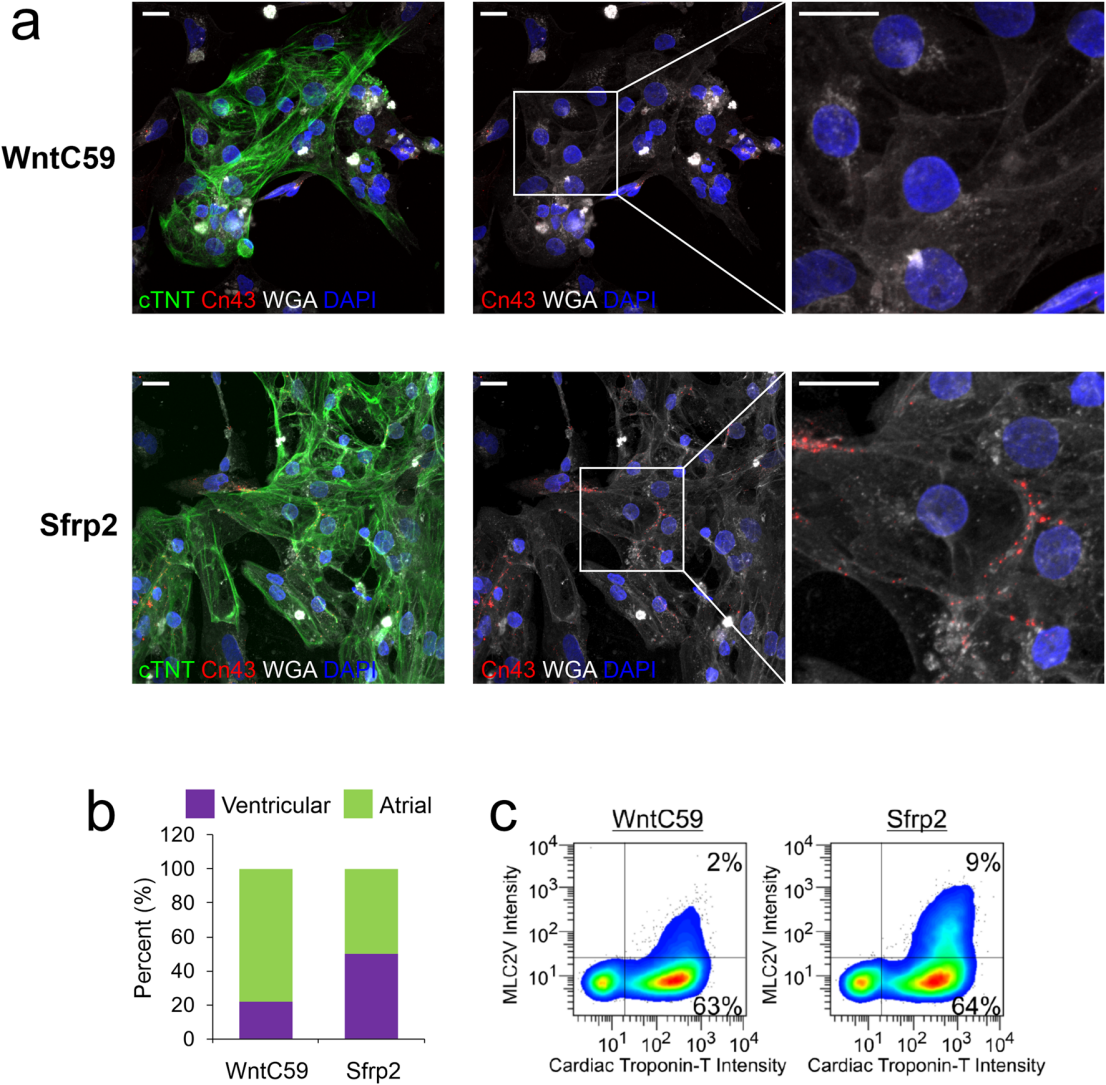
Sfrp2-derived cardiomyocytes form gap junctions. IPSc-derived cardiomyocytes were generated via either the standard broad-spectrum Wnt inhibitor (WntC59) approach or via the Sfrp2 method. After 14 days of differentiation, the cells were analyzed. (**a**) Cells were delineated by staining for the cell membrane marker wheat germ agglutinin (WGA-white). Cardiomyocytes were visualized with cTnT (green) antibodies and nuclei stained with DAPI (blue). Distribution of gap junctions in cardiomyocytes was determined by immunostaining for connexin-43 (red). *N* = 3. Scale bar 20 microns. (**b**) Patch-clamp recordings (Fig. 2e) were analyzed for atrial and ventricular cardiomyocytes. (**c**) Cells were incubated with antibodies targeting the general cardiomyocyte marker cTNT and the ventricular-specific cardiomyocyte marker MLC2v and then subjected to FACS analysis. The graph shows the number of MLC2v + cells as a percentage of the total cardiomyocyte (cTNT +) population. N = 3. Representative traces are shown.

Another hallmark of cardiomyocyte maturation is the formation of gap junctions^21^. Gap junctions are only present in mature cardiomyocytes. In cardiomyocytes generated via the broad-spectrum Wnt inhibitor standard protocol, gap junctions were absent (Fig. 3a, top panels). In contrast, gap junctions were present in cardiomyocytes derived via the Sfrp2 protocol. Importantly, the gap junctions were polarized between cardiomyocytes (Fig. 3a, bottom panels). Polarization is important as this indicates coupling between cardiomyocytes. Taken together, these results indicated that Sfrp2 protocol improves cardiac maturation.

In addition, while Sfrp2 was found to produce both ventricular and atrial cardiomyocytes there was a clear preference for the former (Fig. 3b). This was confirmed by staining for the ventricular isoform of the myosin light chain 2 (MLC2v) (Fig. 3c) and was in contrast to cardiomyocytes generated via the broad-spectrum Wnt inhibitor WntC59.

### Sfrp2 functions as a Wnt3a inhibitor

**Figure 4:**
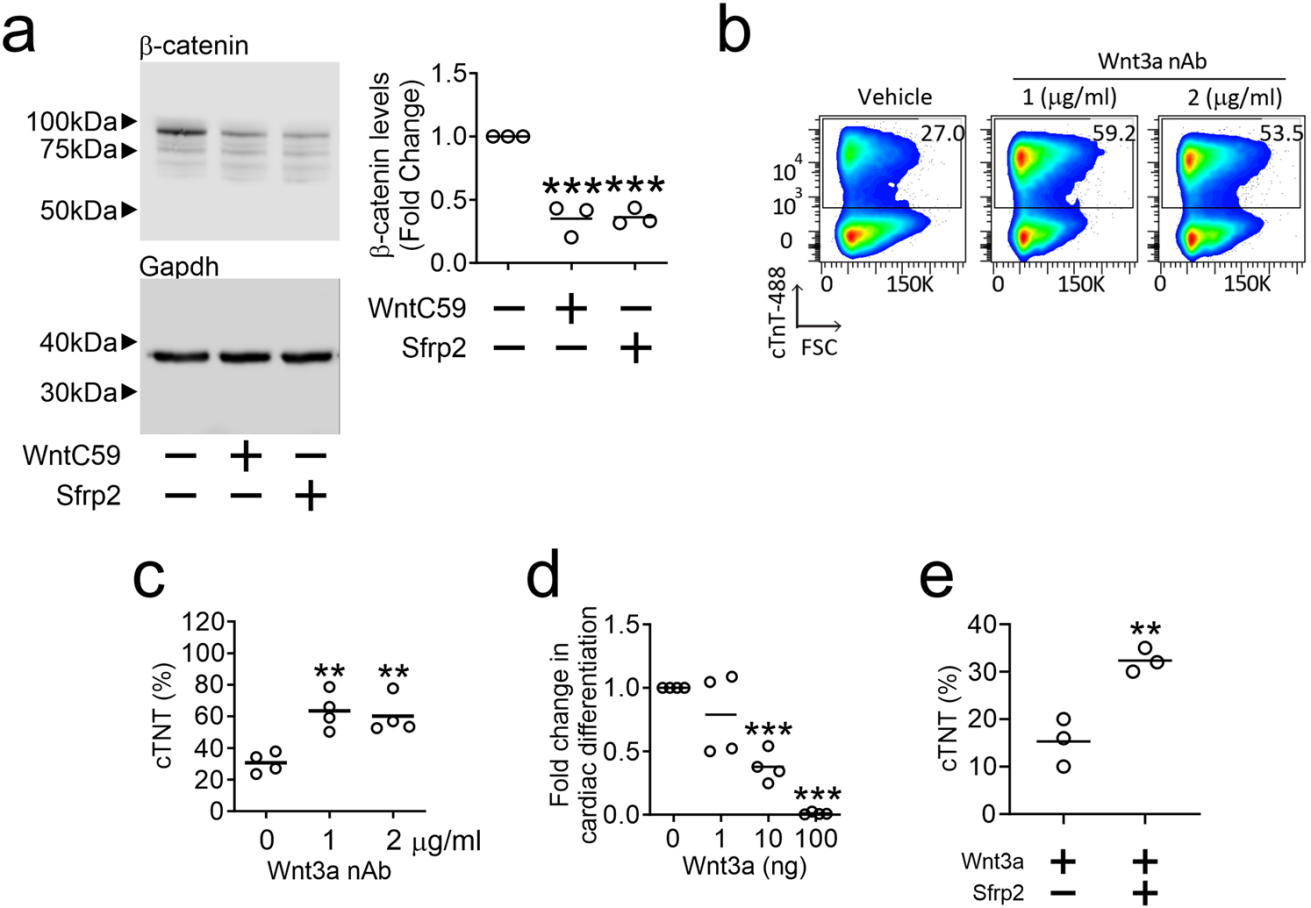
Sfrp2 promotes cardiomyocyte differentiation via of Wnt3a inhibition. (**a**) On day 3 of the differentiation protocol, cells were incubated with either WntC59 (2 μM) or Sfrp2 (1 nM) for 2 days. After two days, cell extracts were analyzed for total-β-catenin by immunoblotting. Total-β-catenin levels were normalized to the housekeeping protein GAPDH and are shown as a fold change compared to the control. Control protein extracts were isolated from cells prior to the addition of WntC59/Sfrp2. Representative immunoblots are shown. *N* = 3. Comparisons are made to control cells: ***P < 0.001. An image showing the immunoblot edge is shown in Supplementary Fig. 1. Note, unlike the images provided here, the image in Supplementary Fig. 1 is non-linear data. As such, it is unsuitable for quantification purposes. (**b & c**) On day 3 of the differentiation protocol, cells were incubated with a human Wnt3a neutralizing antibody (nAb) for 48 h. Cardiomyocyte differentiation was then assessed (day 14) by flow cytometry for cTnT. Representative flow traces are shown in (b) with quantification of the number of cardiomyocytes provided in (c). *N* = 4. Comparisons made to control cells (antibody vehicle): **P < 0.01. (**d**) On day 3 of the differentiation protocol, cells were incubated with WntC59 (2 μm) and the indicated doses of human Wnt3a protein for 48 h. Cardiomyocyte differentiation was assessed (day 14) by flow cytometry for cTnT and the fold change determined by normalizing to the control group (WntC59 treatment and Wnt3a protein vehicle). *N* = 4. Comparisons are made to the control group: ***P < 0.001. (**e**) On day 3 of the differentiation protocol, cells were incubated with human Wnt3a protein (100 ng/ml) and either vehicle or or Sfrp2 (1 nM) for 48 h. Cardiomyocyte differentiation was assessed (day 14) by flow cytometry for cTnT and the number of cardiomyocytes are expressed as a percentage of the total cell population. *N* = 3. Comparisons are made to the control group: **P < 0.01.

We then studied the mechanism underlying the Sfrp2 effect on maturation. Both Sfrp2 and the broad-spectrum Wnt pharmacological inhibitor (WntC59) were found to inhibit the β-Catenin pathway as evidence by reduced total β-Catenin (Fig. 4a). This result suggested that Sfrp2 is potentially influencing cardiac differentiation via a β-Catenin dependent signaling pathway. Previous reports from our laboratory have shown that Sfrp2 influences cell behavior via Wnt3a. Interestingly, Wnt3a neutralizing antibodies were sufficient on their own to induce cardiac differentiation from iPSc (Fig. 4b & 4c). Moreover, the addition of Wnt3a protein was sufficient to inhibit cardiomyocyte differentiation (Fig. 4d). Finally, in iPSc cultured with Wnt3a protein, the addition of Sfrp2 partially rescued cardiomyocyte differentiation when used at their IC50 doses (Fig. 4e). This data indicates that Wnt3a inhibition plays a role in Sfrp2 induced cardiomyocyte differentiation. This data indicates that Wnt3a inhibition plays a role in Sfrp2 induced cardiomyocyte differentiation. However, we cannot formally exclude the possibility that Sfrp2 also influences differentiation via a Wnt-independent pathway.

## Discussion

In conclusion, we demonstrate that substitution of broad-spectrum Wnt pharmacological inhibitors with Sfrp2 improves the standard method of iPSc-derived cardiomyocyte generation by improving cardiomyocyte maturity. Sfrp2 induces cardiac differentiation and maturation potentially via β-Catenin and Wnt3a.

**Figure 5:**
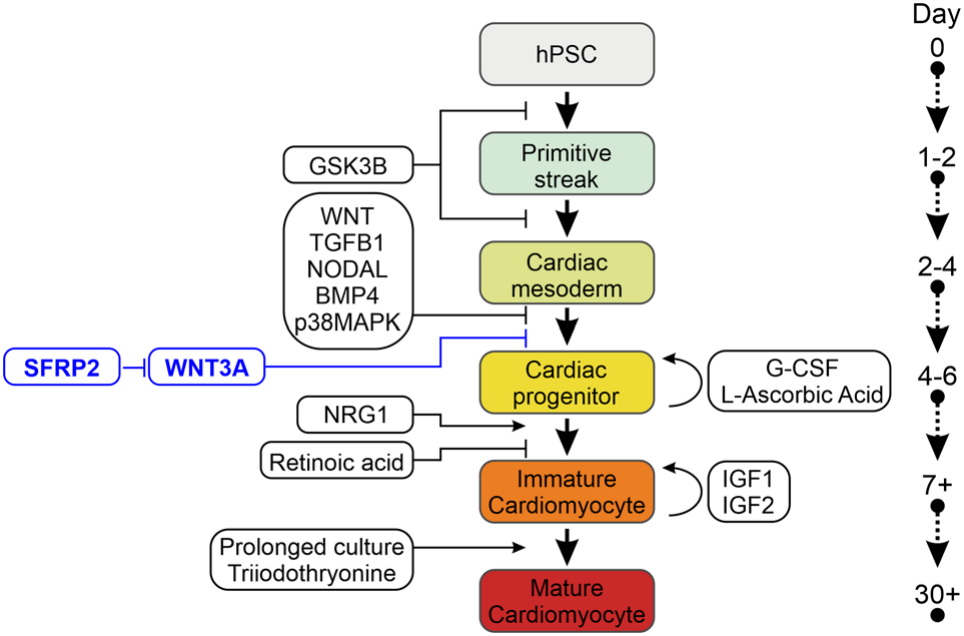
Proposed mechanism of Sfrp2 action. Time-course of iPSc differentiation to mature cardiomyocytes. The colored boxes indicate the various stages of cardiomyocyte differentiation, while the clear boxes indicate the regulatory molecules. The Sfrp2-Wnt3a axis is shown in blue.

There is a substantial body of literature describing the molecular and cellular events during iPSc differentiation to cardiomyocytes (Fig. 5 derived from^22,23^). In the context of what is known, the Sfrp2-Wnt3a axis is placed at the point of cardiac mesoderm specification (Fig. 5). The importance of our finding is specificity. Extant protocols induce cardiac mesoderm specification through the use of broad-spectrum inhibitors. In contrast, our results are more specific as we have identified specific proteins that regulate cardiac mesoderm specification. Our findings with do not exist in a vacuum and other groups have also been successful in promoting cardiomyocyte maturation. Triiodothyronine and glucocorticoid hormones (Fig. 5) increase cardiomyocyte size, anisotropy, and sarcomere length as well as promoting T-tubule formation and electrophysiological maturation^24–26^. Similarly, cardiomyocyte maturation is reported to be improved by the addition of fatty acids to the media^27,28^. It would be interesting to determine the effect of Sfrp2 in these systems.

## Methods

### Generating iPSc-derived cardiomyocytes

Human iPS cells were supplied by Duke University iPSc Core Facility and maintained in mTeSR™ Plus media (Stem Cell Technologies). IPSc-derived cardiomyocytes were generated according to published protocols^6^. The protocol includes a number of reagents with specific roles. Rock inhibitors improve cell survival and plating consistency of the iPScs prior to differentiation. GSK3 inhibitors, such as CHIR99201, are needed for mesoderm specification into cardiac progenitors. The resulting cardiac progenitors can differentiate into several different cell-types. To induce them to differentiate into cardiomyocytes, Wnt/β-catenin inhibitors, such as WntC59 and XAV939, are added to the media. Ascorbic acid is an antioxidant which is known for improving cardiac differentiation. The mechanism by which Ascorbic acid improves the efficacy of cardiac differentiation is unclear. IPSc were seeded at ~ 60% density in 12-well plates coated with Matrigel (Corning, 354,277). One day after seeding (day -3), cells were incubated the Rock inhibitor Y27632 (Tocris, 5 μM) for 24 h in iPSc medium (mTeSR™ Plus media with 50 μg/ml Gentamicin, Thermo Fisher Scientific) and medium every day thereafter with mTeSR™ Plus media. Three days after seeding, the cells were incubated with the GSK3 inhibitor CHIR99201 (Tocris, 6 μM) in differentiation media (RPMI-1640, Thermo Fisher Scientific, with L-ascorbic acid, 100 μg/ml, Sigma) for 24 h. Following the CHIR99201 treatment, the iPSc were incubated with the Wnt inhibitor WntC59 (Tocris, 2 μM), XAV939 (Tocris, 5 μM), human Sfrp2 (R&D systems, 6838-FR-025, 1 nM), human Wnt3a protein (R&D systems, 1324-WN-002), and/or neutralizing anti-human/mouse Wnt3a antibody (R&D systems, MAB9025-100) in differentiation media at day 3. After two days incubation with the agents, the media was replaced with differentiation media from day 5 to day 9. Media was changed on day 7. Day 9 post-seeding, media was changed to RPMI-1640 with B-27™ supplement (Thermo Fisher Scientific) to day 14. Fresh media was added every 2 days.

### Flow cytometry

iPSc-derived cardiomyocytes were removed from the tissue culture plate with Trypsin–EDTA (Thermo Fisher Scientific, 0.25%) and fixed in a solution containing 1% formaldehyde (Thermo Fisher Scientific) and 90% methanol (Sigma). Fixed cells were incubated with antibodies for cardiac troponin-T (Thermo Fisher Scientific, MA5-12,960, 1:200) or MLC2v-PE (Mitenyi Biotec, 130–119-581, 1:50) in FACS buffer (PBS with 0.5% BSA) for 45 min at room temperature. Where necessary, primary antibodies were removed and the cells incubated with an Alexa 488 Goat anti-Ms IgG1 (Thermo Fisher Scientific, A-21121, 1:1,000) in FACS buffer for an additional 30 min at room temperature. Cells were washed three times in FACS buffer and analyzed on a BD Sciences FACSCantoII. Compensation was performed by FlowJo. Singlets were analyzed via gating on the FSC-H and FSC-L channels.

### Immunofluorescence staining and sarcomere analysis

IPSc-derived cardiomyocytes were cultured on glass slides coated with Synthemax II-SC (Corning) in RPMI-1640/B-27 with Y27632 (5 μM) media for 2 days. Cells were fixed with 4% formaldehyde for 15 min at room temperature. Fixed cells were blocked in antibody buffer (5% BSA, 0.1% Tween-20 in PBS) for 1 h at room temperature. Following blocking, cells were incubated overnight at 4 °C with cardiac troponin-T antibody (abcam, ab45932, 1:200), α-Actinin antibody (Sigma, A7811, 1:800), and/or Connexin 43 (Cell Signaling Technology, 1:100) in antibody buffer. After the overnight incubation, cells were washed three times in antibody buffer. Following washing, cells were incubated with Alexa-Fluor conjugated secondary antibodies (Thermo Fisher Scientific, Alexa 488 Donkey anti-Rabbit IgG and Alexa 594 Goat anti-Ms IgG1) at a 1:1,000 dilution in antibody buffer for 1 h at room temperature. Nuclei were stained by DAPI at 1 μg/ml and Wheat Germ Agglutinin (Thermo Fisher Scientific, W21404) was used to stain cell membrane for 10 min at room temperature in antibody buffer. Following washing in PBS to remove unbound complexes, sarcomeres were analyzed using a Zeiss LSM510 confocal microscope. Images were processed with Zeiss software (Axiovision Rel4.8 and Zen Blue). Circularity measurements were made by comparing cardiomyocyte length to width and were expressed as a circularity index whereby circularity index = width/length^15^. Sarcomere distance measurements were made with ImageJ^15^. All measurements were made with a double-blinding method.

### Immunoblotting

Cells were lysed with RIPA buffer containing a Protease-Phosphatase Inhibitor cocktail (Cell Signaling Technology). Proteins (30 μg) were loaded onto 4 to 12% NuPAGE™ gels (Thermo Fisher Scientific) and transferred to nitrocellulose membranes. Membranes were blocked in 5% BSA for 1 h at room temperature. After blocking, membranes were incubated with primary antibodies for β-Catenin and GAPDH in blocking buffer overnight at 4 °C. Primary antibodies were removed by washing in TBS-0.1%Tween20 and membranes incubated with a HRP conjugated anti-rabbit secondary antibody in blocking buffer for 1 h at room temperature. Antibodies all from Cell Signaling Technology and diluted in 1:1,000. After washing the membranes in TBS-0.1%Tween20, bands were visualized via a Amersham ECL Western Blotting Detection kit and signal detected via a Syngene G:BOX. ImageJ was used to quantify band density.

### Patch clamp

IPSc-derived cardiomyocytes were cultured on glass slides coated with BG iMatrix-511 (Biogems, 0.5 μg/cm^2^) in RPMI-1640/B-27 with Y27632 (5 μM) media for 2 days. The microelectrodes (Sutter Instruments, BF150-110-10) were made via a micropipette puller (Sutter Instrument, P-87) to create electrodes at ~ 3 MΩ in Tyrode's solution (140 mM NaC1, 5.4 mM KCl, 1.8 mM CaCl_2_, 1.05 mM MgCl_2_, 0.33 mM NaH_2_PO_4_, 5 mM HEPES and 10 mM glucose; pH was adjusted to 7.4 with NaOH). Current-clamp mode was used to record action potential via an Axopatch 200B amplifier running software Calmpex 8.2.

### Statistics

Except where indicated, all data shown are independent experiments. Statistical analysis was performed using Excel and GraphPad. For groups of two, independent t-tests were performed. For more than two groups, ANOVA was performed with Bonferroni post-hoc tests to determine significance between groups. A P-value of less than 0.05 was considered significant.

## Supplementary Information

Supplementary Information.

## Data Availability

All data generated or analysed during this study are included in this published article.
