# Supplementary material for: Novel method of differentiating human induced pluripotent stem cells to mature cardiomyocytes via Sfrp2
Source: Sci Rep. 2023 Mar 9;13:3920. doi: 10.1038/s41598-023-31144-3 (PMC9998650; doi:10.1038/s41598-023-31144-3)

**Supplementary Figure 1. Immunoblots for Figure 4a.** Syngene software, used for immunoblot imaging, produces several output files which are distinguished by being either linear or non-linear. The image of the luminescence signal is linear. Linear data was used for quantification and for the images provided in Figure 4a. However, as a function of the software, linear data acquisition does not show immunoblot edges. The non-linear data provided by the Syngene software is a composite image combining a black & white image of the blot with an image of the fluorescent signal. This is the only output with an immunoblot edge. It is shown solely at the journal’s request and, as the data is non-linear, it should not be used for any form of quantification.


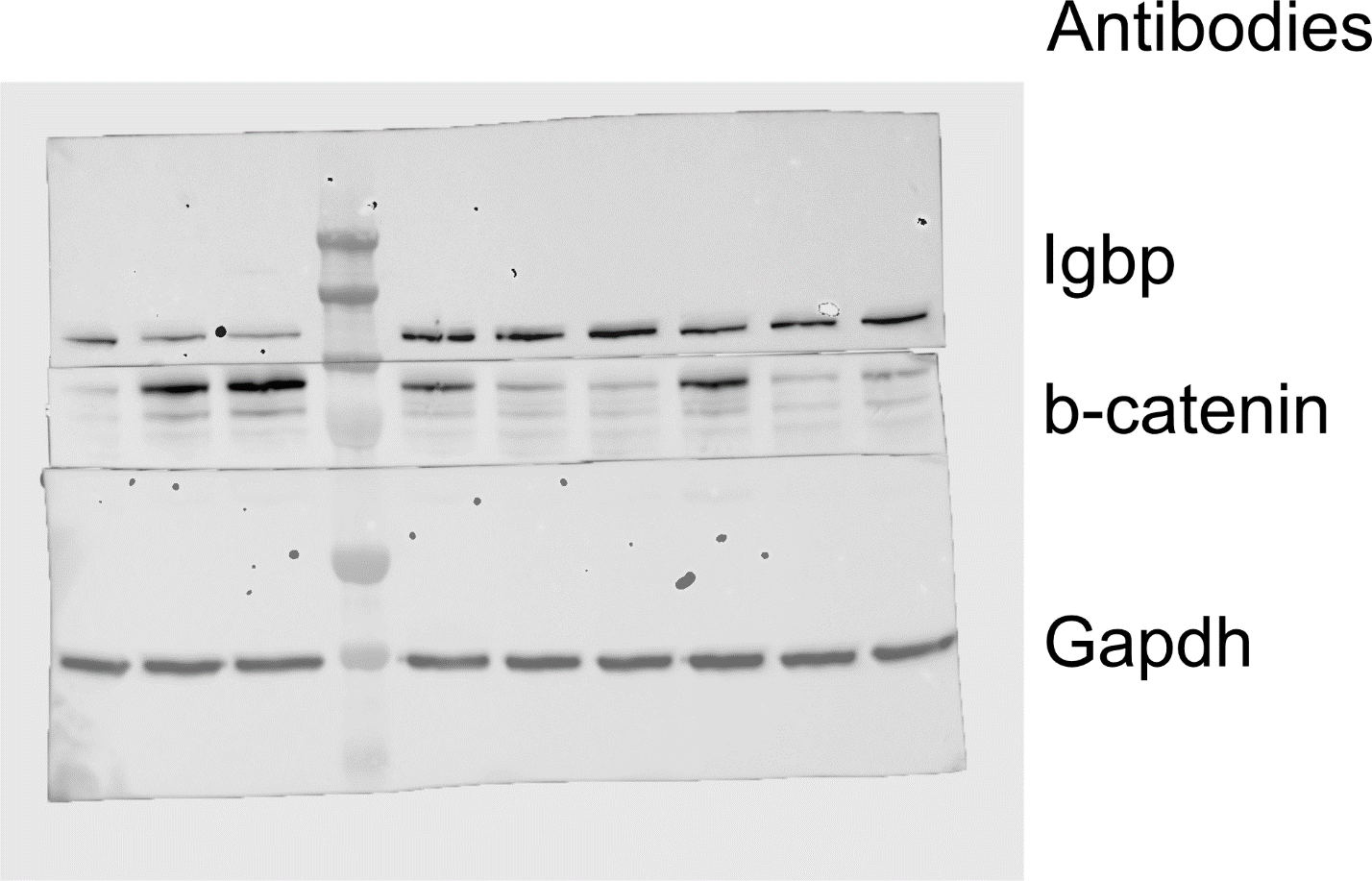

Supplement: Supplementary file 1 — Supplementary Information. [file 41598_2023_31144_MOESM1_ESM.docx]
